# Health Insurance Deductibles and Health Care–Seeking Behaviors in a Consumer-Driven Health Care System With Universal Coverage

**DOI:** 10.1001/jamanetworkopen.2021.15722

**Published:** 2021-07-06

**Authors:** José Luis Sandoval, Dusan Petrovic, Idris Guessous, Silvia Stringhini

**Affiliations:** 1 Unit of Population Epidemiology, Division and Department of Primary Care Medicine, Geneva University Hospitals, Geneva, Switzerland; 2 Department of Oncology, Geneva University Hospitals, Geneva, Switzerland; 3 University Centre for General Medicine and Public Health, University of Lausanne, Lausanne, Switzerland; 4 Centre for Environment and Health, Department of Epidemiology and Biostatistics, Imperial College London School of Public Health, London, United Kingdom; 5 Division and Department of Primary Care Medicine, Geneva University Hospitals, Geneva, Switzerland

## Abstract

**Importance:**

Characteristics of a health care system can facilitate forgoing of health care owing to economic reasons and can influence population health. Whether health insurance deductibles are associated with forgoing of health care in a consumer-driven health care system with universal coverage, such as the Swiss health system, remains to be determined.

**Objective:**

To assess the association between insurance plan deductibles and forgoing of health care with consideration of socioeconomic factors.

**Design, Setting, and Participants:**

This cross-sectional study was conducted in Geneva, Switzerland, using data collected from January 1, 2007, to December 31, 2019. Population-based samples were obtained yearly through random stratified sampling by age and sex of the general population aged 20 to 74 years. Participants were invited to an appointment at 1 of the 3 study sites in Geneva, where they completed a sociodemographic and health questionnaire.

**Exposures:**

Insurance plan deductible level.

**Main Outcomes and Measures:**

The main outcome was forgoing of health care owing to economic reasons. Unadjusted and multivariable Poisson models were used to assess the association between deductible level and forgoing of health care. Differences in forgoing health care across the range of health insurance deductibles or household income levels were quantified using the relative index of inequality (RII).

**Results:**

The study group included 11 872 participants (5974 [50.3%] male; median age, 48.1 years [interquartile range, 38.7-59.1 years]); 1146 (9.7%) reported forgoing health care. Participants with high-deductible plans reported forgoing health care more frequently than those with low-deductible plans (331 [13.5%] vs 591 [8.7%]). In adjusted analysis, higher-deductible plans were associated with a greater likelihood of forgoing health care (RII, 2.2; 95% CI, 1.7-3.0; *P* < .001) independently of socioeconomic status, known comorbidities, and cardiovascular risk factors. Deductible level was associated with forgoing of health care among participants younger than 40 years (RII, 2.5; 95% CI, 1.6-4.0; *P* < .001) and those aged 40 to 64 years (RII, 1.9; 95% CI, 1.3-2.9; *P* = .002) but not among those older than 65 years (RII, 2.9; 95% CI, 0.8-10.4; *P* = .11).

**Conclusions and Relevance:**

In this cross-sectional study, high insurance plan deductibles were associated with forgoing of health care independent of socioeconomic status and preexisting conditions in a universal consumer-driven health care system with good population outcomes in Switzerland. Uncovering health care system design features that could lead to suboptimal population care may help decision makers improve their current health care system design to achieve better outcomes.

## Key Points

**Question:**

Are health insurance deductibles associated with forgoing of health care in a consumer-driven health care system with universal coverage?

**Findings:**

In this cross-sectional study of 11 872 participants in Geneva, Switzerland, between 2007 and 2019, confounder-adjusted analysis revealed that those with higher-deductible plans were significantly more likely to forgo health care than those with low-deductible plans independent of socioeconomic status, known comorbidities, and cardiovascular risk factors.

**Meaning:**

The findings suggest that despite a fixed range of health insurance deductibles established by the government, deductibles may be associated with forgoing of health care in a high-performing universal health care system.

## Teaser

This cross-sectional study examines the association between insurance plan deductibles and forgoing of health care, with consideration of socioeconomic factors, in a consumer-driven health care system with universal coverage in Switzerland.

## Introduction

 Health care expenditures in the US exceed those of comparable nations without producing better outcomes.^1^ Even before the COVID-19 pandemic, changes in the US health care system were deemed unavoidable and the object of intense political debate. US policy makers have attempted to establish a health care system with optimized breadth (population coverage), depth (services covered), and height (proportion of costs covered). The US population tends to favor nongovernment-run health insurance schemes,^2^ and limiting state control of health insurance has been a friction point within and between the leading political parties.

As suggested by Reinhardt,^3^ features of the Swiss health care system, which has a structure slightly similar to that of the US health care system but with better performance, may be of interest to US policy makers. Such features might facilitate bipartisan support for a new health care bill that would improve the current status quo while respecting the US cultural context. The Swiss health care system is an example of a consumer-driven health care system with superior performance, with good population outcomes and universal coverage.^3,4^ The financing of the Swiss health care system relies on mandatory private health insurance with strictly regulated deductibles (US $300, $500, $1000, $1500, $2000, or $2500, with 1 CHF approximately equal to US $1 during the survey period), which are self-selected and independent of preexisting conditions.^5^ Furthermore, medical costs above the deductible are subjected to a 10% co-payment capped at US $700 per year. Individuals with low income can apply for state subsidies to help them pay their premiums. These subsidies are indexed to the insurance premium and the applicant's income level. Dental care is not included in mandatory health insurance plans, and dental insurance is not mandatory and not subsidized by the government.

Although the Swiss health care system has a very high overall performance, it is not free of inequities. Forgoing of health care owing to economic reasons is highly prevalent, with approximately 13.4% of the population reporting it.^6^

Ensuring health equity is one of the major challenges of health care systems, and it is an even greater challenge for those health care systems that are privately funded and have high out-of-pocket expenses. Thus, it is important to identify the health care system features that could constitute potential barriers to achieving horizontal health equity (ie, the same access to health care for individuals with the same need). In this study, we sought to examine the association between the level of insurance plan deductibles and forgoing of health care owing to economic reasons with consideration of socioeconomic factors.

## Methods

### Study Design and Data Collection

In this cross-sectional study, we analyzed data from the Bus Santé study, a repeated health examination survey ongoing since 1993 in Geneva, Switzerland.^7^ Independent samples of Geneva's adult residents (age, 20-74 years) were obtained using a local government's resident list. Random sampling was stratified by sex and 10-year age strata. Bus Santé participants were invited to one of the study sites where they answered standardized questionnaires, and trained collaborators performed general health examinations. Two additional invitations were mailed if contact by telephone was unsuccessful. Participants who could not be reached were classified as nonresponders and were replaced using the aforementioned strategy. All participants gave written informed consent. The Bus Santé study complies with the principles of the Declaration of Helsinki and received approval from the ethics committee of the Geneva University Hospitals. The present study was approved by the institutional review board of the Geneva University Hospitals. This study followed the Strengthening the Reporting of Observational Studies in Epidemiology (STROBE) reporting guideline.

We included data from participants in Bus Santé between January 1, 2007, and December 31, 2019 (years for which data for the outcome and explanatory variables were available). We did not include 2020 data to mitigate the risk of potential bias owing to the COVID-19 pandemic. The mean survey response rate for this period was 51% (range, 47%-56%).

### Measures

We stratified participants according to their insurance plan deductibles (exposure variable): low (US $300/$500), medium (US $1000/$1500), and high (US $2000/$2500). The primary outcome variable, forgoing insured health care owing to economic reasons in the previous 12 months (hereafter referred to as *forgoing health care*), included forgoing care covered by mandatory health insurance: surgical procedures, medical appointments, medication, hospitalization, and home-based care. Forgoing dental health care, which is not covered by mandatory health insurance, was considered separately. No information was available on the number of participants who contracted a private dental insurance plan.

Household income was adjusted to household size using an equivalence scales method: income/(household size)^0.5^.^8^ Tertiles of household income were used when analyses were stratified by this variable. Known comorbidities were self-reported and included a history of myocardial infarction, angina, stroke, or breast cancer. Participants reporting arterial hypertension, diabetes, or dyslipidemia were considered as having cardiovascular risk factors. Age was considered as a continuous variable when included as a confounder in the various statistical models. We considered educational attainment at 3 levels according to the *International Standard Classification of Education 2011*^9^: primary (no professional apprenticeship and no end-of-school certification), secondary (secondary education attained), and tertiary (university degree). Nationality was reported as Swiss or other, and sex, as male or female. Additional variables included having a supplementary nonmandatory health insurance plan and benefiting from subsidized health insurance premiums. The services covered by nonmandatory health plans are varied and determined by the insurance companies. These plans may include, for instance, the use of alternative medicines, travel health insurance, or most commonly, hospitalization in private health care facilities.

Missing data ranged from 0% to 5.9% for all variables except income (25%). For each variable, the proportion of missing data did not differ substantially between groups defined by the explanatory variable (eTable 1 in the Supplement).

### Statistical Analysis

Differences in the outcome and covariates between the groups defined by the exposure variable were tested using χ^2^ and Kruskal-Wallis tests for noncontinuous and continuous variables, respectively. We used unadjusted and multivariable Poisson models to assess the association between deductible level and forgoing health care. The analyses were adjusted for secular trends by including the calendar year as a confounder variable in the statistical models. Interaction terms for age, sex, household income, known comorbidities, and known cardiovascular risk factors were also added to the Poisson models to detect effect modification by these variables. The same models were used to assess the association between household income and forgoing health care.

Differences in forgoing health care across the whole range of health insurance deductibles or household income levels were quantified using the relative index of inequality (RII). This summary measure of inequality has an interpretation similar to that of a prevalence rate ratio between the 2 extremes of the explanatory variable but considers the intermediate groups and relative group sizes.^10^ For instance, an RII of 1.5 would mean a 50% higher prevalence of forgoing health care in the group with high deductibles compared with the group with low deductibles.

We used the RIIGEN package in Stata, version 15.0 (StataCorp LLC)^11^ to calculate exposure variables adjusted for group size and their relative position using a ridit scoring method. The ridit score represents the mean cumulative frequency of each group of an ordinal categorical variable or the midpoint of the range of its cumulative distribution.^12,13^ The ridit scores are sequentially calculated for each group. For instance, if 30% of the population has only primary educational attainment, the ridit score is determined by the following equation: (0 + 0.3)/2 = 0.15. If the next educational level includes 40% of the population, the ridit score for this group would be determined as follows: (0.3 + 0.7)/2 = 0.5. These adjusted variables were then included in Poisson models to estimate the RII. As sensitivity analyses (eTables 2-4 in the Supplement), regression models were survey weighted according to age and sex sampling categories using the 2013 Geneva’s population distribution.^14^

Data were processed and analyzed using Stata, version 15.0 and R, version 3.6.1 (R Project for Statistical Computing). *P* values were 2-sided, and *P* < .05 was considered statistically significant.

## Results

### Study Participants

The study sample included 11 872 participants (5974 [50.3%] male; median age, 48.1 years [interquartile range, 38.7-59.1 years]); 6841 (61.2%) had low-deductible plans, 1875 (16.7%) had medium-deductible plans, and 2454 (22.0%) had high-deductible plans. Overall, 1146 participants (9.7%) reported forgoing health care, and 1399 (11.8%) reported forgoing dental care. Compared with those who had a low-deductible insurance plan, those who had a high-deductible plan were more often male and younger and had higher income and higher educational attainment. Moreover, those with a high-deductible plan less often reported having comorbidities, cardiovascular risk factors, and subsidized insurance premiums. Participants with high-deductible plans reported forgoing health care more frequently (Table 1). Reports of forgoing dental care were similar between groups with different insurance plan deductibles.

**Table 1.** Characteristics of Study Participants by Insurance Deductible Level

Characteristic	Participants^a^				*P* value^c^
	All (N = 11 872)^b^	Deductible level			
		Low (n = 6841)	Medium (n = 1875)	High (n = 2454)	
Sex					
Male	5974 (50.3)	2995 (43.8)	1068 (57.0)	1581 (64.4)	<.001
Female	5898 (49.7)	3846 (56.2)	807 (43.0)	873 (35.6)	
Age, median (IQR), y	48.1 (38.7-59.1)	51.5 (41.2-62.5)	47.4 (39.6-57.2)	42.2 (35.1-50.6)	<.001
Adjusted monthly household income, median (IQR), US $	4763 (3464-6000)	4242 (3000-6000)	5625 (4000-6000)	5625 (4000-6495)	<.001
Educational attainment					
Primary	3068 (26.2)	2069 (30.6)	480 (25.9)	412 (17.0)	<.001
Secondary	3075 (26.2)	1994 (29.5)	421 (22.7)	474 (19.6)	
Tertiary	5583 (47.6)	2695 (39.9)	953 (51.4)	1533 (63.4)	
Swiss nationality					
No	3940 (33.2)	2245 (32.9)	533 (28.5)	788 (32.2)	<.001
Yes	7910 (66.8)	4585 (67.1)	1338 (71.5)	1663 (67.8)	
Known comorbidities					
No	11 196 (94.4)	6312 (92.3)	1813 (96.7)	2403 (98.0)	<.001
Yes	666 (5.6)	525 (7.7)	62 (3.3)	48 (2.0)	
Known cardiovascular risk factors					
No	6956 (58.6)	3518 (51.4)	1210 (64.5)	1762 (71.8)	<.001
Yes	4912 (41.4)	3323 (48.6)	665 (35.5)	692 (28.2)	
Additional nonmandatory insurance plans					
No	6056 (53.5)	3641 (55.1)	861 (47.7)	1300 (55.2)	<.001
Yes	5255 (46.5)	2963 (44.9)	945 (52.3)	1053 (44.8)	
Subsidized insurance premiums					
No	9746 (84.9)	5496 (82.2)	1648 (89.4)	2135 (88.8)	<.001
Yes	1736 (15.1)	1193 (17.8)	196 (10.6)	268 (11.2)	
Forgoing health care (nondental)					
No	10 695 (90.3)	6237 (91.3)	1699 (90.8)	2118 (86.5)	<.001
Yes	1146 (9.7)	591 (8.7)	172 (9.2)	331 (13.5)	
Forgoing dental care					
No	10 470 (88.2)	6026 (88.1)	1659 (88.5)	2145 (87.4)	<.001
Yes	1399 (11.8)	815 (11.9)	216 (11.5)	309 (12.6)	

Abbreviation: IQR, interquartile range.

^a^ Data are presented at number (percentage) of individuals unless otherwise indicated.

^b^ Data were missing for the outcome (forgoing health care) for 31 individuals (0.3%) and for the explanatory variable (insurance plan deductible) for 702 individuals (5.9%).

^c^ *P* values for categorical variables were determined using a χ^2^ test and for continuous variables using a Kruskal-Wallis test.

The distribution of participants according to household income and health insurance deductible level is shown in the Figure, A. The percentage of participants with the highest income level was greater among those with high-deductible plans than among those with low-deductible plans (36% [629 of 1752] vs 27% [1459 of 5386]). Forgoing health care was more frequent among participants with low household income than among those in the highest income group for all health insurance deductible levels (low-deductible plan: 326 of 2110 [16%] vs 75 of 1456 [5%]; medium-deductible plan: 71 of 398 [18%] vs 30 of 419 [7%]; high-deductible plan: 119 of 497 [24%] vs 80 of 628 [13%]) (Figure, B).

**Figure.**
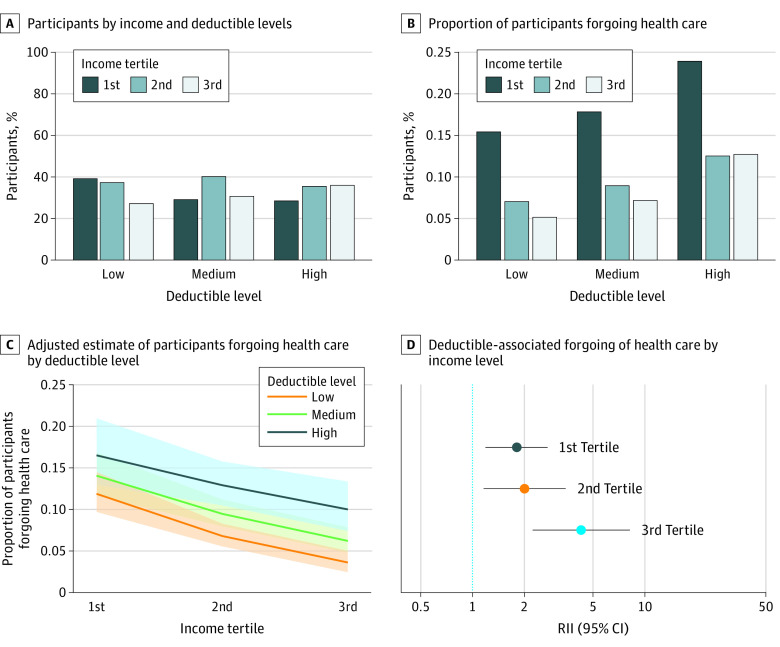
Assessment and Quantification of the Association of Insurance Deductible With Forgoing of Health Care C, Shaded areas represent 95% CIs. D, Error bars represent 95% CIs. For tertile 1, *P* = .005; tertile 2, *P* = .01; tertile 3, *P* < .001. RII indicates relative index of inequality.

Of the 4 most common types of health care that participants reported forgoing (surgical procedures, general practitioner or internal medicine physician appointments, appointments with a specialist, and medication), differences between groups with different deductibles were significant for forgoing appointments with both generalist and specialist physicians (eFigure in the Supplement) but not for forgoing surgical procedures and medication. From 2007 to 2019, the probability of individuals having a high-level deductible plan increased annually (odds ratio from 2007-2019, 1.12; 95% CI, 1.10-1.14, *P* < .001) (eFigure in the Supplement).

### Association of Deductible Level With Forgoing of Health Care

Participants with high-deductible plans were significantly more likely to forgo health care than those with low-deductible plans (RII, 2.0; 95% CI, 1.6-2.6; *P* < .001). The association between deductible level and forgoing of health care was independent of socioeconomic factors defined by income and educational attainment, other sociodemographic characteristics, known comorbidities, and cardiovascular risk factors, with the RII remaining significant after adjustment for these covariates (RII, 2.2; 95% CI, 1.7-3.0; *P* < .001) (Table 2). In contrast, we did not observe an association between deductible level and forgoing of dental health care in both unadjusted (RII, 1.1; 95% CI, 0.9-1.3; *P* = .60) and adjusted (RII, 1.1; 95% CI, 0.8-1.4; *P* = .50) models (eTable 5 in the Supplement).

**Table 2.** Association Between Deductible Level and Forgoing of Health Care

Variable	Unadjusted analysis		Adjusted analysis (n = 7946)	
	Estimate (95% CI)	*P* value	Estimate (95% CI)^a^	*P* value
RII deductible (lowest to highest deductible level)	2.0 (1.6-2.6)	<.001	2.2 (1.7-3.0)	<.001
Adjusted monthly household income	0.8 (0.8-0.8)	<.001	0.9 (0.8-0.9)	<.001
RII of educational attainment (lowest to highest)	0.8 (0.6-0.9)	.009	1.2 (0.9-1.6)	.18
Swiss nationality (vs non-Swiss nationality)	0.8 (0.7-0.9)	.001	1.0 (0.9-1.2)	.66
Sex (female vs male)	1.3 (1.2-1.4)	<.001	1.3 (1.1-1.5)	<.001
Age (per 10 y)	0.8 (0.8-0.9)	<.001	0.9 (0.8-0.9)	<.001
Known comorbidities (vs without)	1.0 (0.8-1.4)	.34	1.1 (0.8-1.5)	.44
Cardiovascular risk factors (vs without)	0.9 (0.8-1.0)	.20	1.1 (1.0-1.3)	.13
Additional nonmandatory insurance plans (vs without)	0.4 (0.4-0.5)	<.001	0.6 (0.5-0.7)	<.001
Subsidized insurance premiums (vs without)	2.2 (1.9-2.5)	<.001	1.5 (1.3-1.8)	<.001
Year of survey (per 5 y)	1.2 (1.1-1.3)	<.001	1.1 (1.0-1.3)	.02
Interactions^b^				
Known comorbidities (vs none)	2.2 (0.7-7.1)	.19	NA	NA
Cardiovascular risk factors (vs none)	0.9 (0.5-1.5)	.68	NA	NA
Sex (female vs male)	0.7 (0.4-1.1)	.14	NA	NA
Household adjusted income	1.6 (1.2-2.3)	.005	NA	NA
Age (per 10 y)	0.8 (0.6-0.9)	.005	NA	NA
Deductibles RII by age category, y^c^				
<40	2.5 (1.7-3.8)	<.001	2.5 (1.6-4.0)	.001
40-64	1.2 (0.9-1.7)	.22	1.9 (1.3-2.9)	.002
>65	1.4 (0.4-4.3)	.61	2.9 (0.8-10.4)	.11

Abbreviations: NA, not applicable; RII, relative index of inequality.

^a^ Results from multivariable Poisson regression assessing the association between insurance plan deductibles and forgoing health care.

^b^ Results from interaction tests between insurance deductibles and the variable of interest in estimating forgoing health of care.

^c^ Results from multivariable Poisson regression assessing the association between insurance plan deductibles and forgoing of health care by age category and including the same covariates as the overall Poisson regression.

### Analysis of Potential Effect Modifiers

The association between deductible level and forgoing of health care was not modified by the presence of known comorbidities (estimate, 2.2; 95% CI, 0.7-7.1; *P* = .19 for interaction), cardiovascular risk factors (estimate, 0.9; 95% CI, 0.5-1.5; *P* = .68 for interaction), or sex (estimate, 0.7; 95% CI, 0.4-1.1; *P* = .14 for interaction). However, we observed a significant effect modification by age (estimate, 0.8; 95% CI, 0.6-0.9; *P* = .005 for interaction); deductible level was associated with forgoing of health care among individuals younger than 40 years (RII, 2.5; 95% CI, 1.6-4.0; *P* < .001) and among those aged 40 to 64 years (RII, 1.9; 95% CI, 1.3-2.9; *P* = .002) but not among those older than 65 years (RII, 2.9; 95% CI, 0.8-10.4; *P* = .11) (Table 2).

### Association of Deductible Level With Forgoing of Health Care in Groups With Different Income Levels and Subsidies

The association between deductible level and forgoing health care was different depending on income level (1.6; 95% CI, 1.2-2.3; *P* = .005 for interaction between deductible level and income). In all tertiles of household income, we observed a significant association between deductible level and forgoing of health care that was more marked among participants with a higher income (tertile 1: RII, 1.8 [95% CI, 1.2-2.7], *P* = .006; tertile 2: RII, 2.0 [95% CI, 1.2-3.5], *P* = .01; tertile 3: RII, 4.3 [95% CI, 2.2-8.2], *P* < .001) (Figure, C and D).

We also assessed whether the association of income level with forgoing of health care varied among individuals with health plans with different deductible levels. The association of income level with forgoing of health care was significant for individuals in all deductible levels but more pronounced for participants with a low deductible (low-deductible plan: RII, 10.1 [95% CI, 6.2-16.5], *P* < .001; medium-deductible plan: RII, 5.9 [95% CI, 2.5-14.0], *P* < .001; high-deductible plan, 4.2 [95% CI, 2.3-7.6], *P* < .001) (eFigure in the Supplement).

Some participants received health insurance–related state subsidies, which might have affected the estimates of forgoing of health care associated with the deductible level. Those with subsidized premiums more frequently had lower deductibles than participants without state aid (1193 of 1657 [72%] vs 5496 of 9279 [59%], *P* < .001).

Deductible level was significantly associated with forgoing of health care among participants with subsidized premiums (RII, 2.1; 95% CI, 1.2-3.7; *P* = .01) and among those without subsidized premiums (RII, 2.3; 95% CI, 1.6-3.2; *P* < .001). Furthermore, among 1025 individuals with a low income and subsidized premiums, we also observed an association between deductible level and forgoing of health care (RII, 2.2; 95% CI, 1.1-4.2; *P* = .02). However, among 1714 individuals with a low income but without a subsidized premium, we did not find an association between deductible level and forgoing of health care (RII, 1.6; 95% CI, 0.9-2.7; *P* = .11).

## Discussion

Health care systems should aim to provide the best available care at the right time while having fair financing and risk protection. We studied the association between deductible level and forgoing of health care in a representative sample of an urban population in Switzerland with a high development index and mandatory nongovernment-run health insurance. As in the US population,^15^ subscriptions to high-deductible plans in the studied population increased over time.

We found that high-deductible plans were associated with an increased probability of forgoing insured health care. Similar results have been observed in the US,^16,17,18,19,20^ where enrollment in high-deductible health insurance plans was associated with decreased health care utilization. In these studies,^16,17,18,19,20^ the reduction in health care utilization affected the entire care continuum including prevention services, outpatient visits, diagnostic tests, chronic disease management, and highly specialized treatments. In the present study, an association of deductible level with forgoing of health care was found for medical appointments and not for medication or surgical procedures, as reported in the US.^17^ The choice of which type of health care to forgo could be, in part, determined by features of the health care system design. A controlled deductibles system could potentially lead individuals to avoid forgoing expensive surgical procedures that exceed their deductible or relatively inexpensive and accessible medication while forgoing intermediate costs such as medical appointments.

In Switzerland, the range of deductible levels is determined by the government to prevent them from being set by a free market, which could leave some individuals without coverage. Mandatory health insurance deductibles can represent between 0.4% and 3.2% of the annual income for a deductible of US $300 or US $2500, respectively (considering a median annual income of US $78 456 in 2018).^21^

Income has been widely described as a primary factor associated with forgoing health care,^22,23,24^ functioning both as a confounder and an effect modifier of deductible-associated differences in forgoing health care. Although the present study was performed in a high-income and human-development setting, in a city that ranks routinely in the top 10 for quality of living conditions worldwide,^25^ social inequalities in forgoing health care were still observed, in line with previous findings.^22^ The association between deductible level and forgoing of health care was identified in all sociodemographic groups but was less pronounced among individuals with low income. These results are in contrast to those of studies in the US that identified inequalities according to deductible level, particularly among individuals with low socioeconomic status.^26,27^

In Geneva, Switzerland, individuals with higher deductibles more often had higher income, probably because deductibles were capped and those with higher income had available capital to cover them when needed. Furthermore, individuals reporting difficulties in paying their insurance premiums because of lack of income can benefit from state subsidies. This additional help may lead those with the lowest incomes to subscribe to plans with low deductibles, relying on state subsidies to pay the higher premiums.

Although we could expect that state subsidies for low-income individuals would eliminate the association of deductible level with forgoing of health care, we observed a significant association between deductible level and forgoing of health care among participants benefiting from subsidies. This finding suggests that subsidized premiums are insufficient to affect the association of deductible levels with forgoing health care.

In contrast to the US health care system, the Swiss system is universal and marked by a higher degree of state subsidization and support.^3^ The findings of this study are thus not generalizable to the US context. Nevertheless, they could help inform US decision makers considering adoption of deductible-related features of the Swiss system in future health care legislation.

### Strengths and Limitations

This study has strengths. The present study was performed in a unique setting. The Swiss health care system mixes private insurance with tight state regulation, allowing the deductible levels to be strictly controlled. The studied population was likely more homogenous than populations of low- and middle-income countries, allowing a better characterization of the association of deductibles with forgoing of health care, which could be masked in national or international studies. In settings where absolute poverty is more prevalent than in Geneva, socioeconomic factors may be more strongly associated with forgoing of health care, preventing the identification of the association between this outcome and insurance deductible level. We analyzed data spanning more than 10 years, excluding the possibility that the identified association was spurious and confined to a specific time point. Furthermore, the use of regression-based inequality indicators allowed the quantification of the association of deductible level with forgoing of health care with consideration of various individual-level factors, including those related to socioeconomic status.

This study also has limitations. First, we assessed forgoing health care owing to economic reasons, but we could not determine the value of forgone care. Although the association between deductible level and forgoing of health care was independent of known comorbidities and cardiovascular risk factors, it remains possible that the value besides the amount of forgone care varied among individuals with different socioeconomic status and deductible levels. However, several studies have shown that high deductibles are associated with reduced necessary and unnecessary health care utilization.^28,29,30,31^ Of note, deductibles in the Swiss health care system apply only to costs incurred in managing diseases and not to costs of accidents. Moreover, the analyzed data were cross-sectional and not longitudinal, and the causal effect of deductible level on forgoing health care could not be determined. Data on the type of forgone medical appointments (eg, first appointments, new episodes of illness, or follow-up) were not available. Also, as in other observational studies, selection bias was possible because of different response rates in population groups with different deductible levels and income. The analyzed period (2007-2019) includes the 2008 economic crisis, potentially impacting both explanatory and outcome variables.

## Conclusions

In this cross-sectional study of a population with high income and a consumer-driven health care system with universal coverage and fixed deductibles, deductible level was associated with forgoing of health care. The association was present at all socioeconomic status levels, suggesting that health insurance deductible level may be associated with forgoing of health care independently of socioeconomic status. Additional studies appear to be needed to assess the association of forgone medical care with health outcomes in this setting. However, because policy makers may be inclined to adopt features of the Swiss health care system, they should be increasingly aware of potential inequities in care associated with health care system design besides those related to individual factors.

## Notes

Supplement. **eFigure.** Type of Forgone Care and Income-Related Disparities in Forgoing Health Care **eTable 1.** Missing Data Details **eTable 2.** Association Between Deductible Level and Forgoing Health Care (Survey-Weighted) **eTable 3.** Association Between Deductible Level and Forgoing Dental Health Care (Survey-Weighted) **eTable 4.** Association Between Deductible Level and Forgoing Health Care in Specific Groups (Survey-Weighted) **eTable 5.** Association Between Deductible Level and Forgoing Dental Health Care
