# Supplementary material for: Health Insurance Deductibles and Health Care–Seeking Behaviors in a Consumer-Driven Health Care System With Universal Coverage
Source: JAMA Netw Open. 2021 Jul 6;4(7):e2115722. doi: 10.1001/jamanetworkopen.2021.15722 (PMC8261614; doi:10.1001/jamanetworkopen.2021.15722)
Supplement: Supplement. — eFigure. Type of Forgone Care and Income-Related Disparities in Forgoing Health Care eTable 1. Missing Data Details eTable 2. Association Between Deductible Level and Forgoing Health Care (Survey-Weighted) eTable 3. Association Between Deductible Level and Forgoing Dental Health Care (Survey-Weighted) eTable 4. Association Between Deductible Level and Forgoing Health Care in Specific Groups (Survey-Weighted) eTable 5. Association Between Deductible Level and Forgoing Dental Health Care [file jamanetwopen-e2115722-s001.pdf]

Supplementary Online Content

Sandoval JL, Petrovic D, Guessous I, Stringhini S. Health insurance deductibles and health care–seeking behaviors in a consumer-driven health care system with universal coverage. *JAMA Netw Open*. 2021;4(7):e2115722. doi:10.1001/jamanetworkopen.2021.15722

eFigure. Type of Forgone Care and Income-Related Disparities in Forgoing Health Care
eTable 1. Missing Data Details
eTable 2. Association Between Deductible Level and Forgoing Health Care (Survey-Weighted)
eTable 3. Association Between Deductible Level and Forgoing Dental Health Care (Survey-Weighted)
eTable 4. Association Between Deductible Level and Forgoing Health Care in Specific Groups (Survey-Weighted)
eTable 5. Association Between Deductible Level and Forgoing Dental Health Care

This supplementary material has been provided by the authors to give readers additional information about their work.

© 2021 Sandoval JL et al. *JAMA Network Open*.

eFigure – Type of forgone care and income-related disparities in forgoing health care

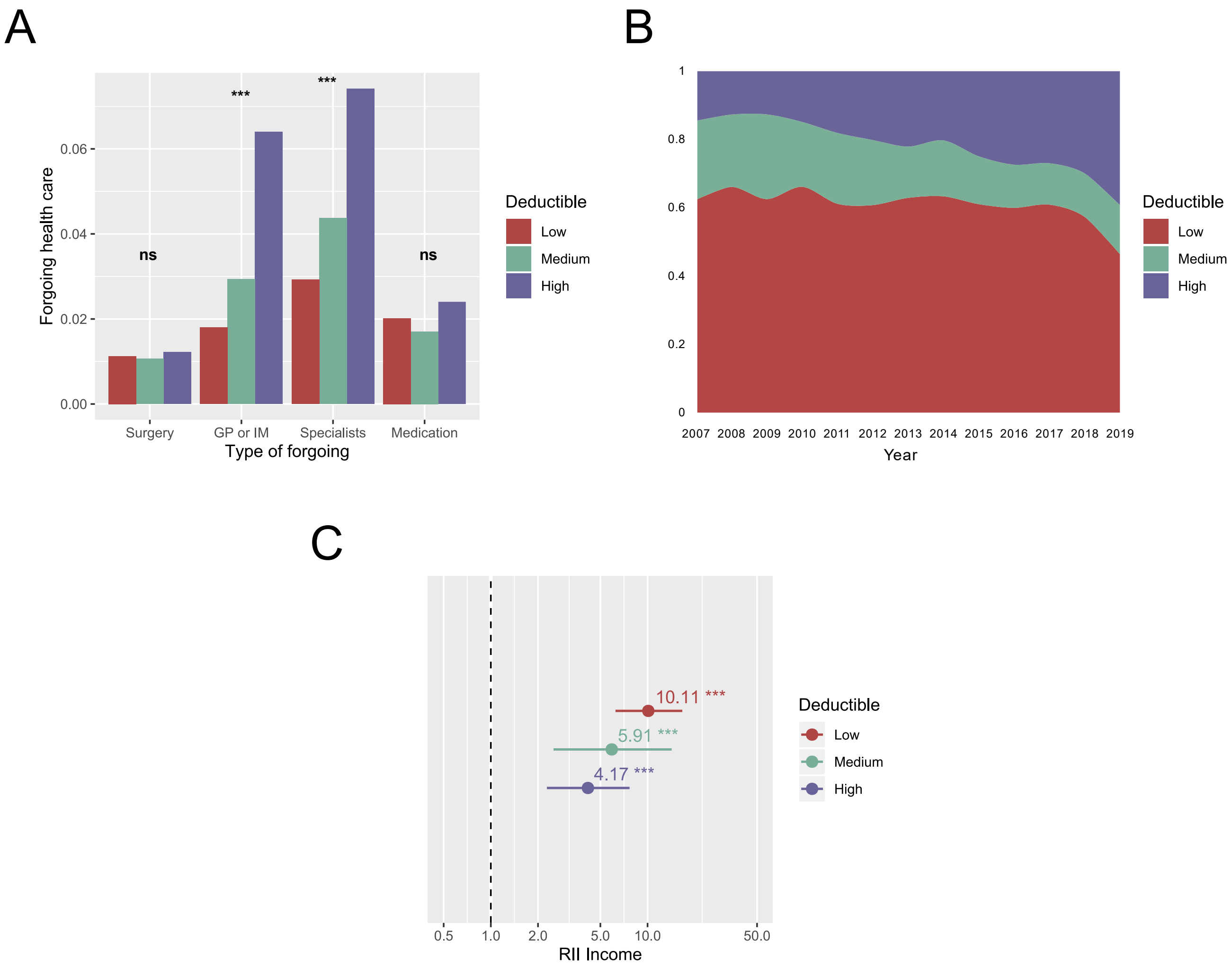

A) proportion of participants forgoing different types of insured care according to deductible level. B) proportion of participants with low, medium or high deductible over time. C) Quantification of income-related health care forgoing according to deductible level. Bars represent 95% CI. RII: relative index of inequality. *p<0.05, **p<0.01, ***p<0.001.

© 2021 Sandoval JL et al. *JAMA Network Open*.

eTable 1 – Missing data details

Variable	Missing data, n (%)			
	Overall	Low deductibles	Medium deductibles	High deductibles
Insurance deductibles	702 (5.91%)	-	-	-
Sex	0 (0%)	0 (0%)	0 (0%)	0 (0%)
Age	0 (0%)	0 (0%)	0 (0%)	0 (0%)
Adjusted household income	2977 (25.08%)	1455 (21.27%)	508 (27.09%)	702 (28.61%)
Educational attainment	146 (1.23%)	83 (1.21%)	21 (1.12%)	35 (1.43%)
Swiss Nationality	22 (0.19%)	11 (0.16%)	4 (0.21%)	3 (0.12%)
Known comorbidities	10 (0.08%)	4 (0.06%)	0 (0%)	3 (0.12%)
Known cardiovascular risk factors	4 (0.03%)	0 (0%)	0 (0%)	0 (0%)
Additional non-mandatory insurance plans	561 (4.73%)	237 (3.46%)	69 (3.68%)	101 (4.12%)
Subsidized insurance premiums	390 (3.29%)	152 (2.22%)	31 (1.65%)	51 (2.08%)
Forgoing health care (non-dental)	31 (0.26%)	13 (0.19%)	4 (0.21%)	5 (0.2%)
Forgoing dental care	3 (0.03%)	0 (0%)	0 (0%)	0 (0%)

© 2021 Sandoval JL et al. *JAMA Network Open*.

eTable 2 – Association between deductible level and forgoing health care (survey-weighted)

	Unadjusted		Adjusted (n=7946)	
	Estimate [95% CI]	p-value	Estimate[a] [95% CI]	p-value
**RII deductible (lowest to highest deductible level)**	2.4 [1.5;3.9]	0.006	2.5 [1.3;4.6]	0.014
**Household adjusted income**	0.8 [0.8;0.9]	<0.001	0.9 [0.8;0.9]	0.001
**RII of educational attainment (lowest to highest)**	0.8 [0.6;1.1]	0.17	1.2 [0.8;1.8]	0.31
**Swiss nationality (vs foreigners)**	0.8 [0.7;0.9]	0.011	1 [0.9;1.1]	0.86
**Sex (vs male)**	1.2 [0.7;2.1]	0.37	1.3 [1.1;1.5]	0.011
**Age (per 10 years)**	0.9 [0.8;0.9]	0.002	0.9 [0.8;0.9]	<0.001
**Known comorbidities (vs without)**	1.1 [0.9;1.2]	0.40	1.2 [0.8;1.6]	0.32
**Cardiovascular risk factors (vs without)**	0.9 [0.8;1.1]	0.30	1.1 [0.9;1.3]	0.2
**Additional non-mandatory insurance plans (vs without)**	0.5 [0.4;0.5]	<0.001	0.6 [0.5;0.8]	0.002
**Subsidized insurance premiums (vs without)**	2.0 [1.9;2.1]	<0.001	1.5 [1.4;1.6]	<0.001
**Year of survey (per 5 years)**	1.2 [1.0;1.4]	0.048	1.1 [0.9;1.3]	0.21
**Interactions**	**Estimate[b] [95% CI]**	**p-value**		
**Known comorbidities (vs without)**	2.6 [0.9;7.1]	0.07		
**Cardiovascular risk factors (vs without)**	0.8 [0.4;1.3]	0.23		
**Sex (vs male)**	0.6 [0.2;2.6]	0.43		
**Household adjusted income**	1.3 [1.1;1.6]	0.013		
**Age (per 10 years)**	0.7 [0.6;0.9]	0.016		
**Deductibles RII according to age categories**	**Estimate [95% CI]**	**p-value**	**Estimate[c] [95% CI]**	**p-value**
**<40 years**	2.9 [0.7;12.8]	0.09	2.8 [0.4;18.2]	0.14
**40-64 years**	1.3 [0.7;2.2]	0.34	1.9 [1.0;3.6]	0.04
**>65 years**	1.4 [0.1;30.5]	0.41	2.9 [0.1;99.7]	0.16

[a] results from multivariable Poisson regression determining the association between insurance plan deductibles and forgoing health care. RII- relative index of inequality, CI – confidence interval

[b] results from interaction tests between insurance deductibles and the variable of interest in predicting forgoing health care

[c] results from multivariable Poisson regression determining the association between insurance plan deductibles and forgoing health care, by age category and including the same covariates as in [a].

© 2021 Sandoval JL et al. *JAMA Network Open*.

eTable 3 – Association between deductible level and forgoing dental health care (survey-weighted)

	Unadjusted		Adjusted (n=7962)	
	Estimate [95% CI]	p-value	Estimate[a] [95% CI]	p-value
**RII deductible (lowest to highest deductible category)**	1.2 [0.8;1.7]	0.24	1.2 [0.9;1.7]	0.13
**Household adjusted income**	0.8 [0.8;0.9]	<0.001	0.9 [0.8;0.9]	<0.001
**RII of educational attainment (lowest to highest)**	0.6 [0.5;0.8]	0.001	1.1 [0.9;1.3]	0.47
**Swiss nationality (vs foreigners)**	0.8 [0.8;1.0]	0.05	1 [0.9;1.2]	0.83
**Sex (vs male)**	1.1 [0.7;1.7]	0.64	1.1 [0.8;1.4]	0.50
**Age (per 10 years)**	0.9 [0.8;0.9]	0.002	0.9 [0.9;0.9]	0.001
**Known comorbidities (vs without)**	1.1 [0.7;1.6]	0.55	1.2 [0.7;2]	0.41
**Cardiovascular risk factors (vs without)**	1.0 [0.9;1.1]	0.44	1 [0.9;1.1]	0.99
**Additional non-mandatory insurance plans (vs without)**	0.4 [0.3;0.5]	<0.001	0.6 [0.4;0.7]	0.003
**Subsidized insurance premiums (vs without)**	1.9 [1.7;2.2]	<0.001	1.3 [1.1;1.5]	0.011
**Year of survey (per 5 years)**	1.2 [1.0;1.4]	0.063	1.1 [1;1.3]	0.12

[a] results from multivariable Poisson regression determining the association between insurance plan deductibles and forgoing dental health care. RII- relative index of inequality, CI – confidence interval

© 2021 Sandoval JL et al. *JAMA Network Open*.

eTable 4 – Association between deductible level and forgoing health care in specific groups (survey-weighted)

	Estimate[a] [95% CI]	p-value
**$RII_{Deductibles}$ in 1st tertile of income**	2.10 [1.1;4.2]	0.04
**$RII_{Deductibles}$ in 2nd tertile of income**	2.0 [1.3;3.1]	0.008
**$RII_{Deductibles}$ in 3rd tertile of income**	4.7 [1.0;21.5]	0.051
**RII_{income} low deductibles**	4.7 [1.6;14.0]	0.015
**RII_{income} medium deductibles**	3.0 [0.9;9.7]	0.061
**RII_{income} high deductibles**	2.3 [0.8;6.7]	0.10
**$RII_{Deductibles}$ in participants with subsidized premiums**	2.5 [1.4;4.8]	0.013
**$RII_{Deductibles}$ in participants without subsidized premiums**	2.3 [1.0;5.4]	0.057
**$RII_{Deductibles}$ in participants with subsidized premiums and low income**	2.5 [0.9;6.8]	0.061
**$RII_{Deductibles}$ in participants without subsidized premiums and low income**	1.9 [0.9;3.9]	0.08

[a] results from multivariable Poisson regression determining the association between insurance plan deductibles and forgoing health care. RII- relative index of inequality, CI – confidence interval

© 2021 Sandoval JL et al. *JAMA Network Open*.

eTable 5 – Association between deductible level and forgoing dental health care

	Unadjusted		Adjusted (n=7962)	
	Estimate [95% CI]	p-value	Estimate[a] [95% CI]	p-value
**RII deductible (lowest to highest deductible category)**	1.1 [0.9;1.3]	0.60	1.1 [0.8;1.4]	0.50
**Household adjusted income**	0.8 [0.8;0.8]	<0.001	0.8 [0.8;0.9]	<0.001
**RII of educational attainment (lowest to highest)**	0.6 [0.5;0.7]	<0.001	1.1 [0.9;1.4]	0.49
**Swiss nationality (vs foreigners)**	0.8 [0.8;0.9]	0.001	1.0 [0.9;1.2]	0.57
**Sex (vs male)**	1.1 [1.0;1.3]	0.03	1.1 [1.0;1.2]	0.22
**Age (per 10 years)**	0.9 [0.8;0.9]	<0.001	0.9 [0.8;0.9]	<0.001
**Known comorbidities (vs without)**	1.1 [0.9;1.4]	0.27	1.2 [1.0;1.6]	0.11
**Cardiovascular risk factors (vs without)**	0.9 [0.8;1.0]	0.19	1.0 [0.9;1.1]	0.96
**Additional non-mandatory insurance plans (vs without)**	0.4 [0.4;0.5]	<0.001	0.6 [0.5;0.7]	<0.001
**Subsidized insurance premiums (vs without)**	2.1 [1.8;2.3]	<0.001	1.3 [1.1;1.5]	<0.001
**Year of survey (per 5 years)**	1.2 [1.1;1.3]	<0.001	1.1 [1.0;1.2]	0.008

[a] results from multivariable Poisson regression determining the association between insurance plan deductibles and forgoing dental health care. RII- relative index of inequality, CI – confidence interval

© 2021 Sandoval JL et al. *JAMA Network Open*.
